# Ringer's lactate improves liver recovery in a murine model of acetaminophen toxicity

**DOI:** 10.1186/1471-230X-11-125

**Published:** 2011-11-15

**Authors:** Runkuan Yang, Shutian Zhang, Henri Kajander, Shengtao Zhu, Marja-Leena Koskinen, Jyrki Tenhunen

**Affiliations:** 1 Department of Critical Care Medicine, University of Pittsburgh Medical School, USA; 2 Department of Gastroenterology, Friendship Hospital, Capital Medical School, China; 3 Department of Pathology, University of Tampere Medical School, Finland; 4 Department of Intensive Care Medicine, University of Tampere Medical School, Finland

## Abstract

**Background:**

Acetaminophen (APAP) overdose induces massive hepatocyte necrosis. Liver regeneration is a vital process for survival after a toxic insult. Since hepatocytes are mostly in a quiescent state (G_0_), the regeneration process requires the priming of hepatocytes by cytokines such as TNF-α and IL-6. Ringer's lactate solution (RLS) has been shown to increase serum TNF-α and IL-6 in patients and experimental animals; in addition, RLS also provides lactate, which can be used as an alternative metabolic fuel to meet the higher energy demand by liver regeneration. Therefore, we tested whether RLS therapy improves liver recovery after APAP overdose.

**Methods:**

C57BL/6 male mice were intraperitoneally injected with a single dose of APAP (300 mg/kg dissolved in 1 mL sterile saline). Following 2 hrs of APAP challenge, the mice were given 1 mL RLS or Saline treatment every 12 hours for a total of 72 hours.

**Results:**

72 hrs after APAP challenge, compared to saline-treated group, RLS treatment significantly lowered serum transaminases (ALT/AST) and improved liver recovery seen in histopathology. This beneficial effect was associated with increased hepatic tissue TNF-α concentration, enhanced hepatic NF-κB DNA binding and increased expression of cell cycle protein cyclin D1, three important factors in liver regeneration.

**Conclusion:**

RLS improves liver recovery from APAP hepatotoxicity.

## Background

Acetaminophen toxicity is the leading cause of drug-induced acute liver failure (ALF) in the US and Europe [1]; however the underlying mechanisms of APAP-induced acute liver injury (ALI) are still not clear. The toxic response to APAP is triggered by a highly reactive metabolite, N-acetyl-p-benzoquinone imine (NAPQI), which reacts with and depletes glutathione (GSH), after which, it forms covalent adducts and initiates mitochondrial oxidative stress [2,3]. This increases the membrane permeability transition and causes the collapse of the mitochondrial membrane potential, which resultantly diminishes the mitochondrial capacity to synthesize ATP [4], and ATP depletion leads to massive necrosis of the hepatocyte, a characteristic feature of APAP hepatotoxicity [5].

Liver regeneration is a vital process for survival after a toxic insult [6,7]. Regeneration ensures the replacement of necrotic cells and the full recovery of organ function. Since hepatocytes are mostly in a quiescent state (G_0_), the regeneration process requires entry into the highly regulated cell cycle [8]. The first step of this process is the priming of hepatocytes by cytokines such as TNF-α and IL-6 [9,10], which makes cells more responsive to growth factors [8]. The exposure to growth factors such as hepatocyte growth factor results in the expression of cell cycle proteins [8]. The induction of cyclin D1 is the most reliable marker for cell cycle (G _1 _phase) progression in hepatocytes [8]. Once hepatocytes express cyclin D1, they have passed the G_1 _restriction point and are committed to DNA replication [8].

Many factors can influence liver regeneration. TNF-α and IL-6 are important pro-regenerative cytokines, which can prime hepatocytes to facilitate liver regeneration [8]. IL-8 and MIP-2, the CXC (CXC motif, the two N-terminal cysteines of CXC chemokines are separated by one amino acid, represented in this name with an "X") pro-inflammatory cytokines, are able to enhance hepatocyte regeneration in acute liver injury induced by APAP even when the treatment is delayed [11]. Currently, NF- κB is thought to play a major role in the initiation of liver regeneration after cell or tissue loss (such as by hepatotectomy) [8]. In addition, nutrients and metabolic status can also influence regeneration, because APAP induces massive hepatocytes necrosis. After the loss of a large number of parenchymal cells, the metabolic work of surviving hepatocytes is increased and more ATP is needed for maintaining homeostasis and regeneration [8].

Ringer's lactate solution (RLS), a frequently used resuscitative fluid, has been shown to increase serum IL-6, IL-8 [12,13] and TNF-α [13,14] in patients and experimental animals; in addition, RLS can provide lactate as an alternative metabolic fuel [15-21]. "Lactic acid" was thought to be responsible for tissue damage, and as a consequence, lactate is frequently considered to be a "toxic" compound. These concepts are now being reexamined as metabolic evidence has emerged in favor of lactate reassessment [22,23]. Lactate provides a satisfactory alternative to glucose as the primary energy in brain tissue during recovery from hypoxia [24,25], and lactate infusion can improve the recovery of neuron damage following brain injury [20]. Moreover, lactate improves cardiac efficiency during shock, and it has recently been shown that lactate deprivation during shock impairs heart metabolism [26]. Such evidence indicates that lactate can be used as an energy substrate and resuscitative fluid to improve liver recovery from APAP-induced hepatotoxicity.

Based on this information, we hypothesize that RLS might improve liver recovery in ALI induced by APAP. To evaluate this idea, ALI was induced in mice by APAP i.p. injection, and the mice were observed over a 72-h period.

## Methods

### Materials

All chemicals were purchased from Sigma-Aldrich Chemical Co. (St. Louis, MO, USA) unless otherwise noted.

### Ethical considerations

This research protocol complied with the regulations regarding the care and use of experimental animals published by the National Institutes of Health and was approved by the Institutional Animal Use and Care Committee of the University of Pittsburgh Medical School (Ref: 0609054A). Male C57BL/6 mice weighing 20-25 g (Jackson Laboratories, Bar Harbor, ME) were used and maintained at the University of Pittsburgh Animal Research Center with a 12-hour light-dark cycle and free access to standard laboratory food and water. The animals were fasted over night prior to the experiments.

### Animal experiments

In the first pilot experiment, 9 mice were intraperitoneally (i.p.) injected with a single dose of APAP (300 mg/kg dissolved in 1 mL sterile saline) and randomized into the RLS group (n = 3), the saline group (n = 3) and the APAP alone group (n = 3). 3 mice injected with saline not containing APAP served as a control group. Following 2 hrs of APAP challenge, each group was empirically given the following treatments every 12 hours for a total of 72 hrs: 0.6 mL RLS for the RLS group, 0.6 mL saline for the Saline group and 0.6 mL saline for the control group (without APAP challenge), no further treatment for APAP alone group. 72 hrs after APAP administration, RLS-treated mice demonstrated significantly lower serum AST than the saline treatment; the saline group had the similar levels of serum ALT/AST as compared with the APAP alone group, suggesting that saline therapy does not have therapeutic effect on APAP-induced ALI. Since no side effect was observed in the RLS group and serum ALT was not markedly reduced by RLS therapy (with a dosage of 0.6 mL), we increased RLS dose from 0.6 mL to 1 mL in the following experiments.

In the second pilot experiment, ALI was induced by a single dose of APAP (300 mg/kg dissolved in 1 mL sterile saline) administered by intraperitoneal (i.p.) injection. APAP challenged mice were then randomized into the RLS (n = 5) group and the Saline group (n = 5). 5 mice injected with saline not containing APAP served as a control group. 2 hrs after APAP administration, each group was given the following treatments every 12 hours for a total of 48 hrs: 1 mL RLS for the RLS group, 1 mL saline for the Saline group and 1 mL saline for the control group (without APAP challenge). 48 hrs after APAP challenge, compared to the saline therapy, RLS treatment statistically decreased serum AST (p < 0.05), however, RLS therapy did not statistically reduce serum ALT concentrations (p > 0.05), therefore, we extended the treatment from 48 h to 72 h in the following experiments.

In the first experiment, ALI was induced by a single dose of APAP (300 mg/kg dissolved in 1 mL sterile saline) administered by i.p. injection. APAP challenged mice were then randomized into the RLS (n = 7) group and the Saline group (n = 7). 6 mice injected with saline not containing APAP served as a control group. 2 hrs after APAP administration, each group was given the following treatments every 12 hours for a total of 72 hrs: 1 mL RLS for the RLS group, 1 mL saline for the Saline group and 1 mL saline for the control group (without APAP challenge). 72 hours after APAP injection, all surviving mice (1 mouse died in each of RLS and Saline groups) in each group were anesthetized with sodium pentobarbital (90 mg/kg i.p.), and the following procedures were performed: 1) blood was aspirated from the heart for the subsequent measurements of aspartate aminotransferase (AST) and alanine aminotransferase (ALT); 2) left lobe of liver was harvested for pathology (HE staining); 3) right lobe of liver was harvested and frozen for following measurements: hepatic NF-κB DNA binding by EMSA; hepatic tissue cyclin D1 expression by Western blot.

In the second experiment, separate 3 groups of mice were treated the same as above except the treatment period was 24 hrs (n = 6 for each group).

### Serum aminotransferase measurements

Serum levels of AST and ALT were measured at 37°C with a commercially available kit (Sigma Diagnostic).

### Histological analysis

Consecutive sections (5 μm) from paraffin-embedded liver were prepared for hematoxylin-eosin staining. The percent of necrosis was estimated by evaluating the number of microscopic fields with necrosis compared with the entire cross section. In general, necrosis was estimated at low power (x100); questionable areas were evaluated at higher magnification (x200 or x400). The pathologist evaluated all histological sections in a blinded fashion. Inflammatory cell infiltration results were scored semi-quantitatively by averaging the number of inflammatory cells per microscopic field at a magnification of 200×. Five fields were evaluated per tissue sample, and six animals in each group were examined.

### Hepatic tissue TNF-α and IL-6 concentrations

To prepare protein extract, we homogenized murine tissue samples with T-PER (Pierce, Rockford, IL), using a 1: 20 ratio of tissue-to-sample preparation reagent, as directed by the manufacturer's instruction. The samples were centrifuged at 10,000 g for 5 min to pellet tissue debris. The supernatant was collected and frozen at -80°C. Protein concentration was determined using a commercially available Bradford assay (Bio-Rad, Hercules, CA). The supernatant was assayed for TNF-α and IL-6 using ELISA kits from R&D Systems (Minneapolis, MN, USA) according to the manufacturer's instructions. The levels were expressed as pg per mg of protein.

### Serum TNF-α and IL-6 concentrations

Blood (1000 μL) was obtained by cardiac puncture and the serum was collected and stored frozen at -80°C until assayed for IL-6 and TNF-α using ELISA kits from R&D Systems (Minneapolis, MN, USA) according to the manufacturer's instructions.

### Tissue myeloperoxidase

Neutrophils infiltration was measured at 72 hours by determining myeloperoxidase (MPO) activity in liver tissue homogenates as previously described [27] and was used as an index of neutrophils infiltration in all groups. The MPO levels were expressed as units per gram of tissue (U/g)

### EMSA

NF-κB activation was determined by EMSA, as previously described [28]. The gels were dried and exposed to Biomax film (Kodak, Rochester, NY) at -70°C overnight with use of an intensifying screen. Bands were scanned at a NucleoVision imaging workstation and quantified with GelExpert release 3.5. Data expressed as Mean ± SEM (n = 6 per condition).

### Western blot

Liver protein was extracted as previously described [29]. Equivalent amounts of protein were boiled in sample buffer and separated on 7.5% pre-cast SDS-polyacrylamide gels (Bio-Rad) and transferred to nylon membranes. Membranes were then probed with a specific antibody against cyclin D1 (Cell signaling Technology, Lexington, KY) protein, visualized with an Enhanced Chemiluminescence substrate (ECL, Amersham Pharmacia Biotech) and exposed to X-ray film according to the manufacturer's instructions.

### Proliferating cell nuclear antigen (PCNA) staining

Sections from mouse liver following APAP challenge were prepared and processed for immunohistochemistry using PCNA staining kits from Invitrogen (Camarillo, CA, USA) according to the manufacturer's instructions.

### Statistical Methods

Results are presented as means ± SEM. Continuous data were analyzed using student's t-test or analysis of variance followed by Fisher's LSD test. P values < 0.05 were considered significant. Summary statistics are presented for densitometry results from studies using western blot for cyclin D1 expression but these results were not subjected to statistical analysis since the method employed was only semi-quantitative (n = 6).

## Results

### Serum ALT/AST at 24 h, 48 h and 72 h time points

**Figure 1:**
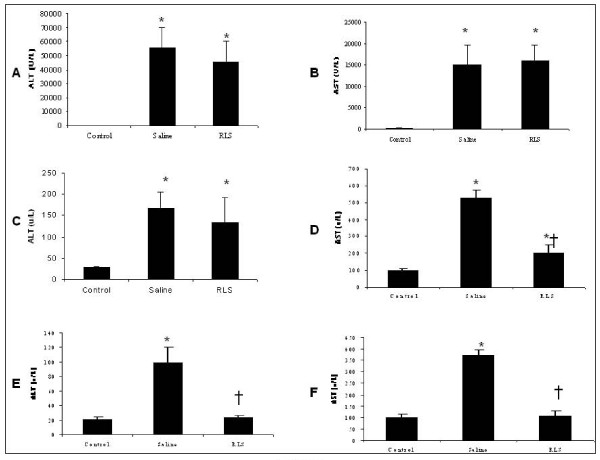
**Effect of treatment with RLS or Saline on ALT/AST in APAP-induced ALI model**. Figure 1A and 1B: ALI was induced in C57Bl/6 male mice with a single dose of APAP (300 mg/kg) by i.p. injection. 2 hrs following APAP injection, the animals were treated with 1 mL RLS or 1 mL saline every 12 hrs. ALT and AST were measured 24 hrs after APAP injection (n = 6 survival mice for each group). Results are means ± SEM. * indicates P < 0.05 versus control. Figure 1C and 1D: three separate groups of mice were used. ALI was induced the same as Figure 1A. 2 hrs after APAP challenge, the animals were given the same treatment every 12 hrs for a total of 48 hrs. ALT and AST were measured 48 hrs after APAP injection (n = 5 mice for each group). Figure 1E and 1F: three separate groups of mice were used. ALI was induced the same as Figure 1A and the same treatment was given every 12 hrs for a total of 72 hrs. ALT and AST were measured 72 hrs after APAP injection (n = 6 survival mice for each group). Results are means ± SEM. * indicates p < 0.05 vs. control; † indicates p < 0.05 vs. saline.

24 hrs after APAP injection, compared to saline treatment group, RLS therapy did not decrease serum concentrations of ALT/AST (Figure 1A, B). 48 hrs after APAP challenge, compared to the saline therapy, RLS treatment significantly decreased serum AST (p < 0.05) but did not statistically reduce serum ALT concentrations (p > 0.05, Figure 1C, D). 72 hrs after APAP challenge, compared to saline treatment group, RLS-treated mice demonstrated significantly lower serum ALT and AST concentrations (* indicates p < 0.05 vs. the control group; †indicates p < 0.05 vs. the saline group) (Figure 1E, F).

### Liver histopathology

**Figure 2:**
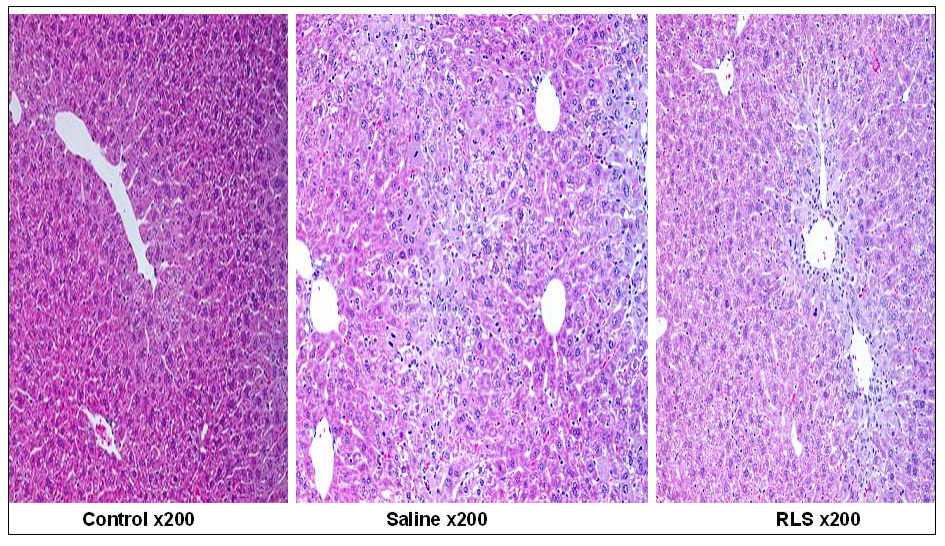
**Effect of treatment with RLS or saline on pathology in mice with ALI**. HE staining was assessed 72 h after induction of ALI (or sham procedure). Method and treatment were the same as described in Figure 1C (n = 6 for each group). Typical picture is shown.

In histological evaluation 72 hrs after ALI induction, compared to control animals, saline-treated mice demonstrated 8.4 ± 1.3% necrotic area, mild regeneration and extensive infiltration of inflammatory cells (260 ± 40 per high power field, n = 6) in the centrilobular region. Loss of cell boundaries and ballooning degeneration were also found around hepatic central vein in the saline group. In contrast, RLS treated mice demonstrated a few scattered individual necrotic hepatocytes and evident regeneration in the centrilobular region; the liver structure has restored to nearly normal. Treatment with RLS did not significantly reduce the number of inflammatory cells (230 ± 35 per high power field, n = 6) (Figure 2).

### Hepatic tissue TNF-α and IL-6

TNF-α and IL-6 are important pro-regenerative cytokines, which can prime hepatocytes at early time point to facilitate liver regeneration, therefore, 24 h time point hepatic tissue TNF-α and IL-6 concentrations were measured. 24 hrs after APAP injection, liver tissue TNF-α levels in the control group and the saline group were undetectable, hepatic tissue TNF- α concentration in the RLS group was 102.2 ± 14 pg/1 mg protein (n = 6). Hepatic tissue IL-6 concentration in the control group was 1800 ± 325 pg/1 mg protein, and this level was not significantly changed in the RLS group or the Saline group (p > 0.05).

### Serum TNF-α and IL-6 concentrations

**Figure 3:**
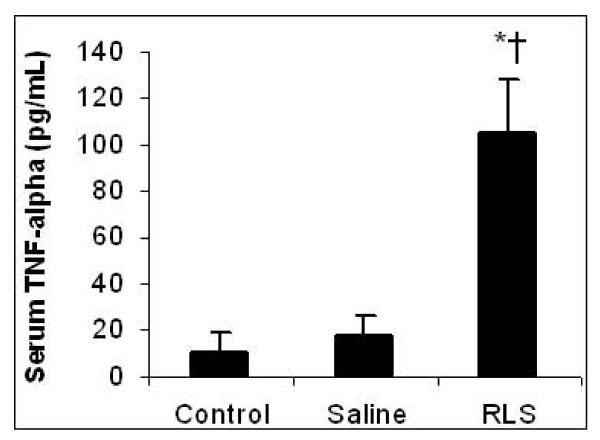
**Effect of treatment with RLS or saline on serum TNF-α in mice with ALI**. Serum TNF-α was assessed 72 h h after induction of ALI (or sham procedure). Results are means ± SEM (n = 6). * indicates p < 0.05 vs. control; † indicates p < 0.05 vs. saline.

24 hours after APAP injection, serum TNF-α concentrations in the RLS and the saline groups remained the same level as in the control group (10.3 ± 8 pg/mL, p > 0.05). However, 72 hrs after APAP administration, compared to saline treatment, RLS therapy significantly increased serum TNF-α concentration (* indicates p < 0.05 vs. the control group; †indicates p < 0.05 vs. the saline group) (Figure 3). Serum IL-6 concentration was undetectable in each group at both 24 h and 72 h time points.

### Hepatic tissue MPO level

Tissue MPO activity was determined as an index of neutrophils infiltration after APAP injection in liver. Liver MPO activity values for the control group were 4.2 ± 0.22 U/g.

**Figure 4:**
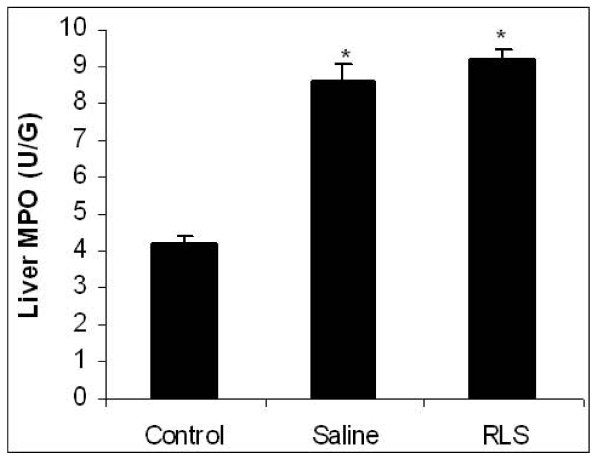
**Effect of treatment with RLS or saline on hepatic MPO activity in mice with ALI**. Liver MPO was assessed 72 h h after induction of ALI (or sham procedure). Results are means ± SEM (n = 6). * indicates P < 0.05 versus control.

72 hours after ALI induction, these values significantly increased to 8.6 ± 0.50 U/g in the saline group and 9.2 ± 0.3 U/g in the RLS group (p < 0.05), but there was no statistical difference between the saline group and the RLS group (n = 6 for each group, data were shown as Mean ± SEM, Figure 4).

### Hepatic NF-κB DNA binding

**Figure 5:**
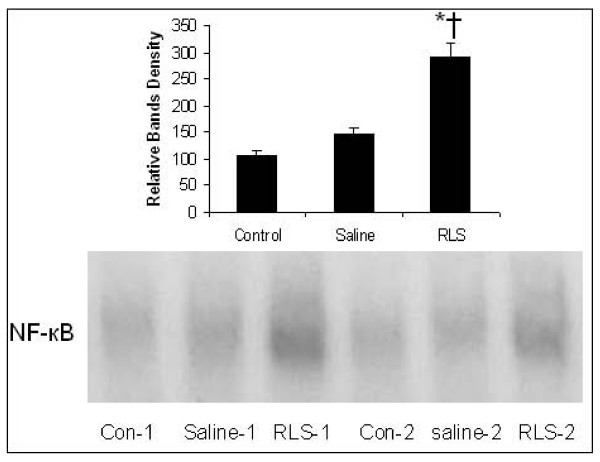
**Effect of treatment with RLS or saline on NF-κB DNA binding in nuclear extracts prepared from hepatic tissue samples from mice with ALI**. NF-κB DNA binding was assessed 72 h h after induction of ALI (or sham procedure). Bands were scanned at a NucleoVision imaging workstation and quantified with GelExpert release 3.5. Data expressed as Mean ± SEM (n = 6 per condition). * indicates P < 0.05 versus Control; † indicates P < 0.05 versus Saline. The figure depicts results from six representative assays (n = 6). Typical gels are depicted

NF-κB is a pleiotropic transcription factor whose activation has been linked to inflammatory and destructive processes, as well as initiation of regenerative programs in the injured liver. Blockade of HMGB1 protects against ischemia-reperfusion (I/R)-induced liver injury; this protection is associated with increased NF-κB DNA binding activity [30]. Enhanced NF-κB activation is seen in mice that are protected from hepatic I/R following blockade of the HMGB1 receptor for advanced glycation end products (RAGE)[31]. Therefore, we examined the impact of APAP on activation of NF-κB at 72 hours after APAP injection and tested the effect of RLS treatment. There was a low basal level of NF- κB DNA binding in the hepatic tissue samples in the control group. In the saline group, there was a slight increase in NF- κB DNA binding. Treatment of mice after APAP challenge with RLS clearly increased NF-κB DNA binding relative to the degree observed in mice treated with saline (Figure 5).

### Hepatic cyclin D1 expression

**Figure 6:**
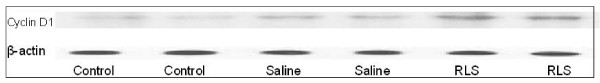
**Effect of treatment with RLS or saline on the expression of cyclin D1 in the hepatic tissue**. Western blot was performed using hepatic extracts prepared from tissues obtained 72 hrs after APAP injection. The figure depicts results from six representative assays (n = 6). Typical gels are depicted.

The timely onset of tissue repair processes can limit liver injury and promote regeneration of lost tissue mass [7]. The induction of cyclin D1 is the most reliable marker for cell cycle (G1 phase) progression in hepatocytes [8]. Western blot was performed using whole-cell extracts prepared from liver tissue to assess expression of cyclin D1 in mice subjected to ALI or the control procedure. In Figure 6, cyclin D1 expression in the control group and saline group was minimal. In contrast, cyclin D1 expression was clearly observed in RLS treated animals at 72 h after APAP administration.

### Hepatic PCNA expression

**Figure 7:**
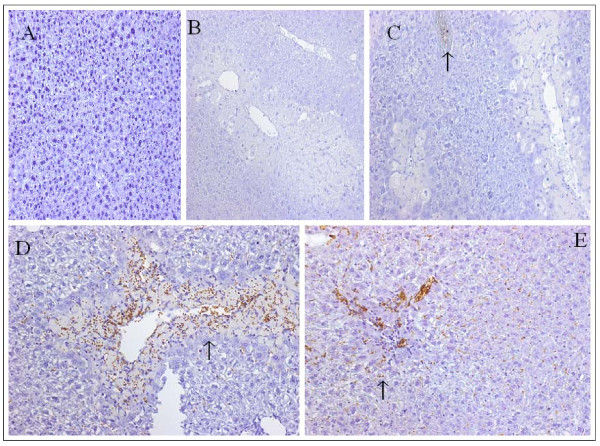
**Effect of treatment with RLS or saline on PCNA in mice with ALI**. PCNA staining was assessed 24 h, 48 h after induction of ALI (or sham procedure). Figure 7A, B and C, the method and treatment were the same as described in Figure 1A and 1B (n = 5 for each group). Figure 7D and 7E: three separate groups of mice were used. ALI was induced the same as Figure 1 A and B. 2 hrs after APAP challenge, the animals were given the same treatment every 12 hrs for a total of 48 hrs. PCNA was detected 48 hrs after APAP injection (n = 5 for each group). Results are means ± SEM. * indicates p < 0.05. Typical picture is shown.

The hepatocyte proliferation was assessed by immunohistological staining for PCNA. The arrows showed PCNA-positive nuclei. At 24 h time point, PCNA was undetectable in the normal control (Figure 7A) or the saline-treated group (Figure 7B), RLS treated group showed 15 ± 2 PCNA-positive nuclei per high power field (Figure 7C) (n = 5 for each group). At 48 hours, the number of labeled nuclei was significantly increased in both saline (Figure 7D) and RLS (Figure 7E) groups, although to a lesser extent in RLS-treated mice. After 48 h, the extent of hepatic PCNA expression, however, depended mainly on the extent of damage, because it was significantly correlated with the area of hepatocyte necrosis for each mouse; our result showed that the saline group had larger necrotic area (25.4 ± 3.7%) than the RLS group (2.2 ± 0.3%), resultantly, the saline group had a larger number of PCNA-positive cells than the RLS group, our result was consistent with Javier Vaquero's report [32]. Additionally, clusters of PCNA-positive cells were seen in the necrotic area, biliary duct and blood vessels in both groups and the number of these clustered cells could not be counted, and most of the PCNA-positive cells were non-parenchyma cells, therefore, the proliferation index could not be calculated. Similar results were seen at 72 h time point; only the number of PCNA-positive nuclei in both groups was less than that in the same treated groups at 48 h (data not shown).

## Discussion

The purpose of this study was to test the hypothesis that RLS treatment improves liver recovery in ALI induced by APAP. The major and the novel findings of this investigation are: (a) RLS-treated mice demonstrate decreased serum ALT and AST and improved liver recovery 72 hrs after APAP administration; (b) the beneficial effect is associated with an augmented NF-κB DNA binding; and (c) RLS-treated mice demonstrate significantly increased expression of cell cycle protein cyclin D1 in liver tissue.

In this study, after RLS treatment, hepatic inflammatory cell infiltration was not reduced as seen in histopathology; neutrophils infiltration was not decreased by measuring MPO activity in total liver extracts. These data indicate that improved liver recovery by RLS therapy is not via changing hepatic inflammatory cells infiltration.

To elucidate the molecular basis of liver recovery in RLS-treated mice, we investigated its effect on the NF-κB signaling pathway because activation of NF-κB is linked strongly not only to the inflammatory response [30], but also to liver regeneration [8]. In addition, NF- κB is currently thought to play a major role in the initiation of liver regeneration after cell or tissue loss (such as partial hepatectomy) [8,10]. NF- κB activation also induces increased expression of survival genes, including BCL_XL _and A1 [33]. Our data suggested that RLS treatment is associated with a beneficial response characterized by activation of NF-κB. Although NF-κB activation modulates inflammation [34], it is also known to protect hepatocytes from cell death, and inhibition of NF-κB after partial hepatectomy results in massive hepatocyte apoptosis, worsens liver injury and decreases survival [35]. Enhanced NF-κB activation is also seen in mice that are protected from hepatic I/R following blockade of the receptor for advanced glycation end products [31]. There is evidence suggesting that the impact of APAP toxicity ensues, at least in part, by dramatic modulation of inflammatory and/or regeneration programs [36]. It is possible that in RLS treated mice subjected to APAP overdose, enhanced NF-κB activation diverts intracellular pathways from those associated with inflammation and cell death to mechanisms linked to recruitment and activation of pro-regenerative programs, therefore, activation of NF-κB by RLS treatment might facilitate regeneration in this ALI induced by APAP.

Massive hepatocyte necrosis is the predominant feature of APAP-induced acute liver injury. Tissue repair is an important determinant of final outcome of toxicant-induced injury [7], and cyclin D1 is an important cell cycle protein. In current investigation, our western blot data showed that RLS treatment markedly increased the level of cyclin D1 in the APAP challenged liver tissue. The change in cyclin D1 expression was associated with decreased serum AST and improved liver regeneration in RLS -treated mice receiving APAP, suggesting that RLS therapy likely facilitates cyclin D1-mediated regeneration pathway, and the increased cyclin D1 expression might be modulated by enhanced NF-κB DNA binding [8].

RLS-treated mice demonstrated lower serum ALT/AST and improved liver recovery at the 72 h time point. This could be due to the increased pro-regenerative cytokine TNF-α, at an early time point by RLS therapy and increased TNF-α, might prime hepatocytes for regeneration. RLS therapy also provides lactate as an alternative metabolic fuel [16-21] to meet the increased energy demand for regeneration after the massive necrosis induced by APAP. Taken together, the increase in pro-regenerative cytokine and in fuel supply might hasten the process of liver recovery from APAP overdose.

In this study, the number of PCNA positive cells was large, and the vast majority of PCNA positive cells were inflammatory cells, this is different from the other studies which have shown hepatocytes at bordering area of necrosis express PCNA during injury progression [37,38]. There are two possibilities for this phenomenon: (1) The specificity and the sensitivity of the first antibody to PCNA might markedly influence the results, because the other two studies used monoclonal antibody to PCNA from DakoCytomation (Carpinteria, CA, USA) and Abcam, (Cambridge, MA), our PCNA staining kit was purchased from Invitrogen (Camarillo, CA, USA). (2) Animal species might also influence the results, because 24 hrs after APAP administration, PCNA expression was apparent in B6C3F1 mice [37] and B6J129 SVF2 mice [38], however, PCNA staining was negative or occasional in C57/BL6 mice in our study at 24 h time point, and our result was consistent with another study [32] in which BrdU (5-bromo-2-deoxyuridine) immunohistochemistry was performed to detect hepatocyte regeneration in C57/BL6 mice.

Currently NAC, a glutathione precursor, is the antidote for APAP overdose [39]. However, this antidotal therapy is effective for early-presenting patients, and is less effective for late-presenting patients [39,40], additional therapies are needed. Treatment with RLS would serve as an adjuvant to NAC therapy. Further experiments designed to compare the two therapies are needed in the future.

Since RLS treatment is able to improve liver recovery from APAP overdose in which massive necrosis of the hepatocyte is a characteristic feature, it is possible that RLS therapy may improve hepatic regeneration following liver surgery (such as partial hepatectomy). Further study is needed to confirm this idea in the future.

## Conclusion

RLS treatment improves liver recovery after APAP overdose and RLS may present a novel therapy to treat APAP hepatotoxicity.

## Abbreviations

APAP: acetaminophen; ALI: acute liver injury; RLS: ringer's lactate solution; I.P. intraperitoneal; ATP: Adenosine triphosphate; NAPQI: N-acetyl-p-benzoquinone imine; TCA: tricarboxylic acid; ALT: alanine aminotransferase; AST: aspartate aminotransferase; MPO: myeloperoxidase; NF-κB: nuclear factor κB; GSH: glutathione; RAGE: receptor for advanced glycation end products; EMSA: electrophoretic mobility shift assays; NAC: N-acetyl-cysteine

## Competing interests

The authors declare that they have no competing interests.

## Authors' contributions

RKY designed the study. All authors participated in the animal handling and procedures. All authors read and approved the final manuscript.

## Pre-publication history

The pre-publication history for this paper can be accessed here:

http://www.biomedcentral.com/1471-230X/11/125/prepub
